# Case Report: When reflux smells like smoke: a case of phantosmia associated with gastroesophageal reflux

**DOI:** 10.3389/fgstr.2026.1867837

**Published:** 2026-10-08

**Authors:** Christopher Visak, Skylar Ketteler, Shireen Husami, Imran Shafqat

**Affiliations:** 1 Western Michigan University Homer Stryker MD School of Medicine, Kalamazoo, MI, United States; 2 Bronson Healthcare Family Medicine, Kalamazoo, MI, United States

**Keywords:** case report, gastroesophageal reflux disease, olfactory dysfunction, phantosmia, proton pump inhibitor

## Abstract

**Background:**

Phantosmia is the perception of an odor without an external stimulus. Gastroesophageal reflux disease (GERD) has been associated with olfactory dysfunction, but phantosmia has rarely been described.

**Case presentation:**

A 40-year-old woman with longstanding GERD developed intermittent perceptions of cigarette smoke during a week of worsening heartburn and bloating. Episodes were accompanied by déjà vu, nausea, and chills. A double-contrast barium esophagram demonstrated a small hiatal hernia and reflux to the thoracic inlet. She reported approximately 80% improvement after starting omeprazole, with brief recurrence during a heartburn flare, and complete resolution at later follow-up. Migraine with aura, allergic rhinitis, focal seizures, and psychiatric causes were considered as alternative explanations.

**Conclusion:**

The close relationship between reflux activity and olfactory symptoms, together with improvement during reflux-directed treatment, supports a plausible association between GERD and phantosmia. This single case does not establish causation, and neurologic and sinonasal etiologies should remain part of the evaluation.

## Introduction

Phantosmia is a qualitative olfactory disorder characterized by perception of an odor in the absence of an external stimulus. Commonly recognized causes include sinonasal disease, migraine, focal seizures, psychiatric conditions, medications, and intracranial pathology (1, 2). Because olfactory hallucinations may reflect neurologic or structural disease, these possibilities should be considered during evaluation. Gastroesophageal reflux disease (GERD) and laryngopharyngeal reflux have also been associated with impaired olfactory threshold, discrimination, and identification, possibly through reflux-related inflammation of the upper airway and nasal mucosa (3–5). However, most published work concerns quantitative olfactory impairment rather than phantom odor perception. We present a case of episodic phantosmia that began during a GERD exacerbation, fluctuated with heartburn, and resolved during reflux-directed therapy.

## Case presentation

### Patient information and chief complaint

A 40-year-old woman presented with three days of intermittent, intense perceptions of cigarette smoke. The episodes occurred while awake and around sleep and lasted up to a few hours. She also reported a strong sensation of déjà vu accompanied by nausea and chills. The episodes occurred during routine activities and were not associated with reported loss of consciousness. During the preceding week, she had developed worsening heartburn and bloating that began before the phantosmia and were most pronounced in the morning. She also described colicky abdominal pain and reduced appetite. The symptoms were distressing, particularly because she was preparing for an international move.

### Past medical history and medications

The patient reported GERD since adolescence. An upper endoscopy at age 23 reportedly showed incomplete closure of the lower esophageal sphincter, and previous weight loss had improved her reflux. Her history also included chronic allergic rhinitis, migraine with aura, anxiety, and depression. A brain MRI obtained in 2021 for chronic unilateral headache showed a 7-mm pineal cyst without mass effect and a tiny nonspecific left frontal subcortical FLAIR hyperintensity, interpreted as possibly migraine-related or due to chronic small-vessel change. At presentation, she was using fluticasone nasal spray and fexofenadine-pseudoephedrine for allergic rhinitis but was not taking a proton pump inhibitor (PPI).

### Physical examination

She was afebrile, with blood pressure 112/70 mmHg, heart rate 62 beats/min, and respiratory rate 18 breaths/min. She appeared well. Abdominal examination demonstrated minimal tenderness and right lower quadrant hypertympany, with negative Murphy and McBurney signs. Nasal examination showed bilateral turbinate hypertrophy with moderate airway crowding. Subsequent examinations documented that she was alert and oriented, with coherent speech, logical thought processes, appropriate behavior, and no focal neurologic deficit. A PHQ-9 score of 15 had been recorded on August 4, 2025.

### Diagnostic workup

Helicobacter pylori breath testing was negative, TSH was normal, and abdominal radiography showed minimal ileus with mild-to-moderate fecal loading. Because her bloating and nausea were associated with fatty foods and she had a prior gallbladder ejection fraction of 10%, the gastrointestinal evaluation focused on repeat hepatobiliary imaging as well as reflux. The repeat study on September 18, 2025, showed patent ducts and a normal ejection fraction of 74%. A gastric-emptying scan was not obtained; therefore, delayed gastric emptying was not formally excluded.

A double-contrast barium esophagram on September 24, 2025, demonstrated a small hiatal hernia and gastroesophageal reflux to the thoracic inlet. The esophagus otherwise distended and contracted normally, without reported mass, stricture, or mucosal abnormality. Upper endoscopy was recommended, but the patient was hesitant and did not complete it. Formal olfactory testing and ambulatory reflux monitoring were not performed.

On November 9, 2025, after the olfactory symptoms had begun, the patient slipped and fell down several stairs. A noncontrast head CT obtained the following day showed no acute intracranial abnormality, although mild motion artifact was present. The CT was obtained for trauma rather than as a targeted evaluation of phantosmia and was not used to exclude epilepsy. No contemporaneous brain MRI, EEG, or neurologic consultation was documented.

### Treatment and clinical course

Omeprazole was initiated for worsening reflux. Follow-up prescriptions and the subsequent medication list documented a dose of 40 mg each morning, taken approximately 30 minutes before breakfast. She was also counseled to avoid dietary triggers and late meals, elevate the head of her bed, and pursue weight loss. Her existing allergic rhinitis treatment with intranasal fluticasone and an oral antihistamine continued; the records did not document a new or intensified rhinitis regimen when omeprazole was started.

At approximately one month, the patient reported an 80% decrease in the olfactory symptoms. During the two days before that visit, both heartburn and the phantom smoke smell briefly returned, and she became more careful about avoiding reflux triggers. At a later follow-up, approximately three months after presentation, she reported complete resolution of the olfactory symptoms while continuing omeprazole and improving her sleep hygiene and weight-related behaviors. Reflux symptoms were also controlled. The outcomes were patient-reported; no formal repeat olfactory testing was performed. The clinical timeline is summarized in **Table 1**.

**Table 1:** Clinical timeline.

Time	Clinical event
February 2021	Brain MRI for chronic unilateral headache showed a small pineal cyst without mass effect and a tiny nonspecific left frontal FLAIR focus.
Week before presentation	Heartburn and bloating worsened before onset of the olfactory symptoms.
Three days before presentation	Intermittent cigarette-smoke perceptions began, accompanied by déjà vu, nausea, and chills.
Initial evaluation	Examination, negative H. pylori test, normal TSH, and abdominal radiography; omeprazole and reflux lifestyle measures initiated.
Approximately 1 month	Patient reported 80% improvement; brief recurrence of both heartburn and phantosmia during the preceding two days.
September 18, 2025	Repeat hepatobiliary scan showed normal gallbladder emptying.
September 24, 2025	Double-contrast barium esophagram showed a small hiatal hernia and reflux to the thoracic inlet.
Approximately 3 months	Patient reported resolution of phantosmia while continuing omeprazole; reflux was controlled.
November 9-10, 2025	Fall occurred after olfactory symptom onset; trauma-related head CT showed no acute intracranial abnormality.

### Patient perspective

The patient perceived a close connection between worsening heartburn and the smell of cigarette smoke and adjusted her diet to avoid triggers. She reported meaningful improvement in appetite and quality of life as the reflux and olfactory symptoms subsided, which reduced her concern about maintaining symptom control during her upcoming move.

## Discussion

This case describes episodic phantosmia arising during a flare of longstanding GERD and resolving during reflux-directed therapy. Several features support a clinically meaningful association: heartburn and bloating preceded the olfactory symptoms, objective imaging demonstrated reflux to the thoracic inlet, the patient reported substantial improvement after starting omeprazole, and both heartburn and phantosmia briefly recurred together before later resolving. This pattern does not prove causation, but it is more informative than improvement alone and supports the plausibility of reflux as a contributor.

Prior studies have shown impaired olfactory threshold, discrimination, and identification in patients with GERD, as well as correlations between reflux severity and subjective taste or smell disturbance (3, 4). A recent scoping review concluded that the evidence remains limited and heterogeneous but identified several studies supporting an association between laryngopharyngeal reflux and olfactory dysfunction (5). A prospective study published subsequently found improvement in measured olfactory function after 12 weeks of PPI therapy in patients with laryngopharyngeal reflux (6). These studies do not specifically establish a relationship with phantosmia, which is what makes the present observation unusual.

A reflux-mediated mechanism is biologically plausible. Proximal refluxate may expose the upper airway and nasal mucosa to acid and pepsin, particularly when supine, contributing to local inflammation and altered olfactory signaling (5, 6). In this patient, symptoms were most prominent in the morning and the esophagram demonstrated reflux reaching the thoracic inlet. However, the study did not establish laryngopharyngeal exposure, and no objective olfactory or reflux-monitoring measurements were obtained. The clinical response therefore supports, but cannot confirm, this mechanism.

Neurologic alternatives warrant specific consideration. Olfactory hallucinations, déjà vu, nausea, and chills can occur as focal temporal lobe phenomena (1, 2). In this case, the reported hours-long duration was less typical of a brief focal aura, and symptoms resolved without antiseizure treatment. However, loss of consciousness is not required for focal seizures, and episode stereotypy, automatisms, post-event symptoms, and preserved responsiveness were not systematically documented. The older MRI and later trauma CT do not exclude epilepsy or subtle structural disease, and no EEG or neurologic consultation was completed. Migraine-associated phantosmia also remains possible given her history of migraine with aura, although her usual headaches occurred later in the day and no consistent relationship between headache and the olfactory episodes was documented (7).

Allergic rhinitis is another potential confounder because turbinate hypertrophy was present. Importantly, intranasal corticosteroid and antihistamine therapy were already listed at presentation rather than newly initiated with omeprazole. The timing of improvement therefore aligned more closely with initiation of reflux therapy, although unrecorded changes in adherence or nasal symptoms prevent exclusion of a sinonasal contribution. Anxiety and depression were also present, but follow-up examinations documented organized thought and behavior without psychotic features. Psychiatric consultation was not obtained, so a psychiatric contribution cannot be fully excluded. Medication-related, postinfectious, and other neurologic or anatomic causes were not identified in the available history.

The gastrointestinal differential also included delayed gastric emptying because of bloating and nausea. The clinician prioritized reflux and biliary evaluation based on prominent heartburn, fatty-food symptoms, and the previous abnormal gallbladder study. Repeat hepatobiliary imaging was normal, but this does not assess gastric emptying. The absence of gastric-emptying scintigraphy is therefore a limitation rather than evidence against gastroparesis (8).

The principal limitations are the single-patient design, subjective symptom reporting, lack of formal olfactory testing and ambulatory reflux monitoring, incomplete neurologic and psychiatric evaluation, and the presence of concurrent lifestyle and rhinitis therapies. The initial omeprazole schedule could not be independently verified beyond the documented follow-up dose of 40 mg each morning, and longer recurrence-free follow-up was unavailable. Spontaneous resolution also cannot be excluded. Despite these limitations, the close temporal relationship between reflux and phantosmia, including their brief simultaneous recurrence, makes the association worthy of further study.

## Conclusion

This case suggests that GERD may be an underrecognized and potentially reversible contributor to phantosmia in selected patients. The observed course does not establish causation, and focal seizures, migraine, and sinonasal disease remain important alternative explanations. When phantosmia occurs alongside a clear reflux exacerbation, treatment of documented GERD may be reasonable while the need for neurologic, otolaryngologic, or other evaluation is guided by the clinical presentation. PPI therapy and lifestyle measures are recommended for management of typical GERD symptoms (9).

## Data Availability

The original contributions presented in the study are included in the article/Supplementary Material. Further inquiries can be directed to the corresponding author.
